# Clinical- and cost-effectiveness of the STAR care pathway compared to usual care for patients with chronic pain after total knee replacement: study protocol for a UK randomised controlled trial

**DOI:** 10.1186/s13063-018-2516-8

**Published:** 2018-02-21

**Authors:** Vikki Wylde, Wendy Bertram, Andrew D. Beswick, Ashley W. Blom, Julie Bruce, Amanda Burston, Jane Dennis, Kirsty Garfield, Nicholas Howells, Athene Lane, Candy McCabe, Andrew J. Moore, Sian Noble, Tim J. Peters, Andrew Price, Emily Sanderson, Andrew D. Toms, David A. Walsh, Simon White, Rachael Gooberman-Hill

**Affiliations:** 1 Musculoskeletal Research Unit, Translational Health Sciences, Bristol Medical School, University of Bristol, Learning and Research Building, Southmead Hospital, Bristol, BS10 5NB UK; 2 National Institute for Health Research Bristol Biomedical Research Centre, University Hospitals Bristol NHS Foundation Trust and University of Bristol, Bristol, UK; 3 North Bristol NHS Trust, Bristol, UK; 4 Warwick Clinical Trials Unit, University of Warwick, Warwick, UK; 5 Bristol Randomised Controlled Trials Collaboration, Population Health Sciences, Bristol Medical School, University of Bristol, Bristol, UK; 6 Department of Nursing and Midwifery, Faculty of Health and Applied Sciences, University of the West of England, Bristol, UK; 7 Nuffield Department of Orthopaedics, Rheumatology, and Musculoskeletal Science, University of Oxford, Oxford, UK; 8 Exeter Knee Reconstruction Unit, Royal Devon and Exeter Hospital, Exeter, UK; 9 Arthritis Research UK Pain Centre and NIHR Nottingham BRC, University of Nottingham, Nottingham, UK; 10 Cardiff & Vale Orthopaedic Centre, University Hospital Llandough, Penarth, UK

**Keywords:** Total knee replacement, Chronic post-surgical pain, Care pathway, Randomised controlled trial

## Abstract

**Background:**

Approximately 20% of patients experience chronic pain after total knee replacement. There is little evidence for effective interventions for the management of this pain, and current healthcare provision is patchy and inconsistent. Given the complexity of this condition, multimodal and individualised interventions matched to pain characteristics are needed. We have undertaken a comprehensive programme of work to develop a care pathway for patients with chronic pain after total knee replacement. This protocol describes the design of a randomised controlled trial to evaluate the clinical- and cost-effectiveness of a complex intervention care pathway compared with usual care.

**Methods:**

This is a pragmatic two-armed, open, multi-centred randomised controlled trial conducted within secondary care in the UK. Patients will be screened at 2 months after total knee replacement and 381 patients with chronic pain at 3 months postoperatively will be recruited. Recruitment processes will be optimised through qualitative research during a 6-month internal pilot phase. Patients are randomised using a 2:1 intervention:control allocation ratio. All participants receive usual care as provided by their hospital. The intervention comprises an assessment clinic appointment at 3 months postoperatively with an Extended Scope Practitioner and up to six telephone follow-up calls over 12 months. In the assessment clinic, a standardised protocol is followed to identify potential underlying causes for the chronic pain and enable appropriate onward referrals to existing services for targeted and individualised treatment. Outcomes are assessed by questionnaires at 6 and 12 months after randomisation. The co-primary outcomes are pain severity and pain interference assessed using the Brief Pain Inventory at 12 months after randomisation. Secondary outcomes relate to resource use, function, neuropathic pain, mental well-being, use of pain medications, satisfaction with pain relief, pain frequency, capability, health-related quality of life and bodily pain. After trial completion, up to 30 patients in the intervention group will be interviewed about their experiences of the care pathway.

**Discussion:**

If shown to be clinically and cost-effective, this care pathway intervention could improve the management of chronic pain after total knee replacement.

**Trial registration:**

ISRCTN registry (ISRCTN92545361), prospectively registered on 30 August 2016.

**Electronic supplementary material:**

The online version of this article (10.1186/s13063-018-2516-8) contains supplementary material, which is available to authorized users.

## Background

Treatment of osteoarthritis with total knee replacement aims to reduce pain, functional limitations and associated disability. Over 100,000 primary total knee replacements were performed in the United Kingdom (UK) in 2015 [1, 2]. Despite good outcomes for many, a systematic review found that approximately 20% of patients report chronic pain after total knee replacement [3]. Chronic post-surgical pain is defined as pain that occurs or increases in intensity at 3 months or longer after surgery [4]. Patients with bothersome pain at 3 months after surgery are often disappointed with their outcome [5, 6], feel abandoned by healthcare [7] and struggle to make sense of ongoing pain [8]. Chronic pain after knee replacement is an under-investigated area, but the wider literature shows the impact of chronic pain on all areas of life. Chronic pain is associated with poor general health, interference with daily activities, disability and depression [9–11]. Compared with the general population, patients with chronic musculoskeletal pain report lower satisfaction with life [12–14]. Older people with pain are likely to become socially isolated, which is a risk factor for other problems [15], limiting their capacity to bring about change or to seek help for their pain.

Healthcare provision for patients with chronic pain after total knee replacement has been shown to be patchy and inconsistent in the UK, with only some orthopaedic centres having standardised protocols to guide the assessment and management of patients with this condition [16]. A systematic review identified that only one trial has evaluated an intervention for the management of chronic pain after knee replacement – an injection with antinociceptive and anticholinergic activity [17]. There is also insufficient evidence about the effectiveness of interventions for the management of chronic pain after any surgery type [18]. Therefore, there is a need for robust evidence to guide the early screening, identification and management of patients with chronic pain after total knee replacement.

Treatment of chronic pain is challenging, and evaluation of treatments in combination or matched to patient characteristics is advocated [19], yet no such trials have been evaluated in the context of chronic post-surgical pain [18]. It has been argued that rather than new interventions for pain, improvements are required to access existing treatments with combination treatments matched to pain characteristics [19]. Chronic pain after total knee replacement may be caused by biological and mechanical factors. Biological causes include the sensitising impact of chronic pain from osteoarthritis [20–22], development of Complex Regional Pain Syndrome [23–25], persistent postoperative inflammation, infection and/or localised nerve injury [26]. Mechanical causes include altered gait, prosthesis loosening, and weakening effects on ligaments [27, 28]. Psychological factors may also influence postoperative outcomes [29].

To improve the management of chronic pain after total knee replacement, we have developed the STAR (Support and Treatment After joint Replacement) care pathway [30], which consists of early postoperative screening to identify patients with pain and an assessment clinic at 3 months postoperatively with an Extended Scope Practitioner and telephone follow-up, as required. The intervention aims to enable appropriate onwards referral to existing services to ensure that underlying reasons for chronic pain are considered early in the postoperative pathway and that treatment is targeted at these to improve pain management and to reduce the impact of pain. In line with UK Medical Research Council guidance on complex interventions, comprehensive development work has been undertaken to design and refine this intervention. The design of the intervention is underpinned by a systematic review [17], survey of current practice [16], focus groups with health professionals [31], expert deliberation and patient involvement activities [32]. Further development and refinement work included consensus work with health professionals to refine intervention content, testing intervention delivery and acceptability to patients, and evaluation of views about implementation of the intervention within the context of a randomised controlled trial [30]. The aim of this multi-centre randomised controlled trial is to evaluate the clinical and cost-effectiveness of the care pathway for patients with chronic pain after total knee replacement.

## Methods/Design

**Fig. 1:**
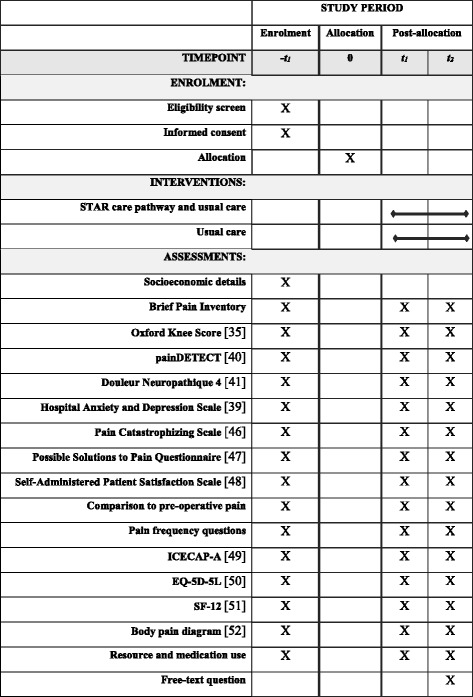
SPIRIT Figure for the schedule of enrolment, interventions and assessments

This protocol follows guidance from SPIRIT (Standard Protocol Items: Recommendations for Interventional Trials) [33, 34]. A SPIRIT figure for the schedule of enrolment, interventions and assessment is provided in Fig. 1 and a SPIRIT checklist is provided in Additional file 1.

### Aim

The primary aim of this trial is to evaluate the clinical effectiveness of a new care pathway (‘the STAR pathway’) when compared with usual care for people with chronic pain after knee replacement. Secondary objectives of embedded aspects of the trial include:

1. Pilot phase with qualitative work to optimise recruitment and refine trial processes;

2. Economic analysis to evaluate the cost-effectiveness of the care pathway;

3. Qualitative study with patients who received the intervention to explore their experiences of the care pathway.

### Design

Core trial information are presented in Appendix – WHO Trial Registration Data Set. This is a pragmatic, parallel, two-arm, superiority, multi-centred randomised controlled trial using 2:1 intervention:control randomisation, with an internal pilot phase and embedded economic evaluation and qualitative studies. The trial is currently taking place at four high-volume National Health Services (NHS) centres for total knee replacement, and will be expanded to include 8–10 trial sites.

### Regulatory approvals

Ethics approval was obtained from South West – Central Bristol Research Ethics Committee in July 2016 (REC reference 16/SW/0154) and HRA approval in August 2016. Any protocol amendments will be submitted to the HRA for approval prior to implementation and updated on the ISRCTN registry.

### Patient involvement in trial design

Patients were involved in trial design through the University of Bristol’s Musculoskeletal Research Unit’s ‘Patient Experience Partnership in Research’ (PEP-R) patient involvement groups [32]. The PEP-R Musculoskeletal group comprises nine patients with musculoskeletal conditions, most of whom have had joint replacement. The PEP-R STAR group is a specialised group established for this programme of work, comprising five patients with experience of chronic pain after knee replacement. Both of these groups inputted into trial design, acceptability of randomisation, design of data collection and primary outcomes, questionnaires, patient information leaflets, recruitment consultations and qualitative topic guides.

### Patient recruitment

**Fig. 2:**
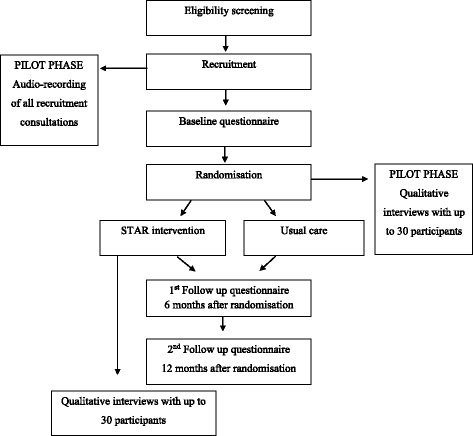
Participant flow through the trial

A diagram of participant flow in the trial is provided in Fig. 2.

#### Eligibility criteria

Inclusion criteria are adults aged ≥ 18 years who have received a primary total knee replacement because of osteoarthritis at a participating NHS Trust and who report pain in their operated knee at 2–3 months after surgery, assessed using the 7-item pain subscale of the Oxford Knee Score (OKS) [35] (each item on the OKS is scored 0–4, with a total pain score of 0–28, severe pain to no pain). Based on previous cluster analysis [36], patients with pain are defined as those with a score of 0–14 on the OKS pain subscale.

Exclusion criteria are a lack of capacity to provide informed consent to participate, previous participation in the STAR trial for the contralateral knee, or participation in another research study that interferes unacceptably with the STAR trial.

#### Screening process to identify patients with chronic pain after knee replacement

An NHS employee will search hospital computer systems to identify all patients who had received a primary total knee replacement for osteoarthritis 2 months previously. These individuals will be sent a pre-screening notification card; 2–4 days later, they will be sent a screening study pack. Anonymised data on the age and sex of all patients sent a screening pack will be recorded. The screening pack includes a cover letter, patient information leaflet, screening questionnaire, a freepost envelope and a complimentary teabag. One reminder screening pack will be sent if no response is received within 1–2 weeks. Patients are asked to complete and return the screening questionnaire and consent form. On receipt of a completed screening questionnaire, the research team scores the OKS to identify patients with pain in their replaced knee (score of 0–14 on the OKS pain subscale).

#### Recruitment process

Patients who score 0–14 on the screening OKS pain subscale and consent to further contact from the research team will be posted a trial information pack and then telephoned by a researcher 3–5 days later. If participating, they will then complete a second OKS via telephone with the researcher to ensure they still meet the inclusion criteria for the trial. A face-to-face recruitment consultation at the participant’s home or local hospital is then arranged. Some of the final detailed aspects of this recruitment consultation will be informed by work during the pilot phase of the trial and will follow a model consultation process [37]. If a patient would like to participate in the trial, they will be asked to provide informed, written consent. All patients will be provided with a sheet of publicly available contact details for relevant charities or organisations, such as Arthritis Care, Pain Concern and Mind. Participants are then given a baseline questionnaire to complete and return to the research team. All researchers involved in recruitment have Good Clinical Practice and trial-specific training.

### Randomisation

After participants have provided written, informed consent and have returned a completed baseline questionnaire, they will be randomly allocated to the STAR pathway or usual care. Participants will be informed of their allocation by a member of the research team. Randomisation occurs as soon as possible after the baseline questionnaire is received. Randomisation with allocation concealment is conducted remotely via the Bristol Randomised Trials Collaboration using a web-based randomisation system. Randomisation takes place on a 2:1 basis to ensure that the intervention service is running at sufficient capacity to enable a pragmatic assessment of its clinical and cost-effectiveness. Moreover, if the intervention is operating to a sufficient degree of capacity, then per-protocol and Complier Average Causal Effect analyses will be more reliable and have higher power. To ensure reasonable balance between the two treatment groups, allocation is minimised by pain severity and pain interference scores for the replaced knee (assessed with the Brief Pain Inventory Severity and Interference Scales and categorised into tertiles based on data from a previous study [30]), and stratified by trial centre.

Blinding of participants and trial personnel to treatment allocation is not possible due to the nature of the intervention. After participants have been randomised, the research team will send the participant and their General Practitioner (GP) a letter to inform them of treatment allocation.

### Usual care

All patients in the trial receive usual care as provided by their hospital. The trial sites all provide a routine 6-week postoperative follow-up, and one centre provides an additional 3-month appointment. All centres provide additional follow-up with a surgeon if requested but do not include routine follow-up by practitioners specialising in pain.

### Intervention

**Fig. 3:**
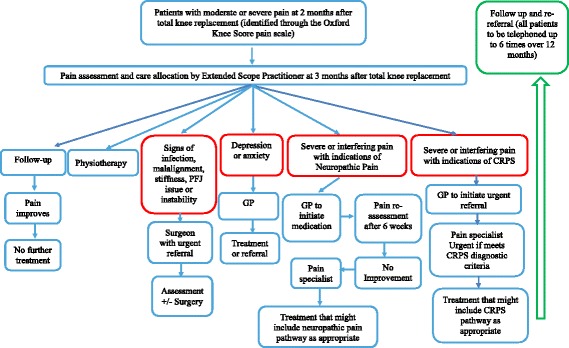
Schematic overview of STAR care pathway

Participants randomised to the intervention group will receive usual care and the STAR intervention, which consists of a 1-hour-long assessment clinic appointment with a trained Extended Scope Practitioner (a registered allied healthcare professional with specialist training in orthopaedics) and up to six telephone follow-up calls over 12 months (Fig. 3). Adherence to the intervention is defined as attendance at the assessment clinic appointment. Participants will be offered an assessment clinic appointment as soon as possible after randomisation, ideally within 1 week. Booking an appointment is arranged over the telephone and confirmed by letter.

The clinic appointment is booked for 1 hour and involves the Extended Scope Practitioner taking a clinical history, reviewing patient-reported outcome measures, conducting a knee examination, and reviewing radiographs and blood test results. Patient-reported outcome measures include the Brief Pain Inventory (BPI) [38], Hospital Anxiety and Depression Scale (HADS) [39], painDETECT [40] and Douleur Neuropathique 4 (DN4) [41]. The knee examination involves evaluating the sites and nature of knee tenderness, surgical wound healing, range of motion, alignment, stability, patellofemoral joint function, signs of infection, and signs and symptoms of Complex Regional Pain Syndrome as per the Budapest criteria [42]. A blood sample is taken to test for markers of infection. Participants have anteroposterior long leg alignment, lateral, and patella skyline knee radiographs taken if these have not already been performed as part of their usual care to evaluate alignment and assess for evidence of fracture or concerns with sizing, fixation or position of the implants. The appointment may last longer than 1 hour since additional time is required for radiographs.

Findings from the assessment clinic appointment are recorded on a standardised proforma and entered into the research database. On the basis of the STAR assessment, participants are referred to appropriate existing services for further treatment, which may include one or more of the following: a surgeon, when pain is attributable to surgical factors; physiotherapy for exercise and mobility advice and support; a GP for treatment of depression or anxiety; and/or pain specialists for neuropathic pain or Complex Regional Pain Syndrome (via GPs). Monitoring is also available if this is appropriate. The STAR care pathway is individualised and flexible, and other referrals can be made depending on the needs of the participant. Copies of all referral letters are sent to the patient, their treating orthopaedic surgeon and their GP.

Participants receive telephone follow-up from the Extended Scope Practitioner based on clinical need, up to a maximum of six times over 12 months. These telephone calls are to follow-up on the received and to ensure that any referrals are being undertaken. Additionally, further referrals can be made on the basis of these telephone follow-up consultations. Details of these telephone calls and any additional referrals made after the follow-up telephone call are documented on a standardised proforma.

All Extended Scope Practitioners delivering the intervention attend a 1-day training session and are provided with a comprehensive intervention training manual that includes standard operating procedures for the assessment. Further details of the development and content of the intervention, in line with the template for intervention description and replication (TIDieR) [43], has been published separately.

### Minimisation of contamination

It is possible that evolution of usual care over time may be influenced by implementation of the intervention at participating sites. To minimise this risk, we will liaise with the Principal Investigators and key trial staff at each site to ensure that information about the trial is disseminated to local clinical staff, taking care that this is not counterproductive. The provision of usual care will be monitored through resource use questions in the follow-up questionnaires at 6 and 12 months after randomisation.

### Co-treatments

Participants can seek treatment for related or unrelated medical conditions as needed during the trial. Use of health services are recorded in follow-up questionnaires and will be used in the health economics analysis.

### Assessment of intervention fidelity

Intervention fidelity evaluates the degree to which an intervention is delivered as intended [44]. In this trial, assessment clinics and telephone follow-up calls will be observed to evaluate if the intervention is being delivered as intended in the intervention training manual. A minimum of one assessment clinic for each Extended Scope Practitioner involved in intervention delivery will be observed annually. Observations are recorded on a standardised proforma and any additional training needs are highlighted and actioned.

### Outcome measurement

All participants are assessed at baseline prior to randomisation (3 months after surgery) and at 6 months (9 months after surgery) and 12 months (15 months after surgery) after randomisation. All outcome measurements will be undertaken via self-report questionnaires and participants are provided with a complimentary teabag with each questionnaire. Participants are offered the option of completing study questionnaires on paper or online through REDCap (https://www.project-redcap.org/). If completed questionnaires are not received within 2 weeks, a reminder questionnaire will be sent. If no response is received to the reminder, a researcher will telephone the participant to offer support in completing the questionnaire on the telephone. Telephone calls to participants who do not return a follow-up questionnaire will be performed by a researcher from a different trial centre to ensure that the researcher is blinded to treatment allocation.

The primary and secondary outcomes map directly onto the eight domains of the core outcome set for the assessment of chronic pain after knee replacement [45]. Details of the time point for each outcome are provided in Fig 1. The co-primary outcomes are pain severity and pain interference assessed using the BPI [38] at 12 months after randomisation. Participants will be asked to complete the BPI in relation to their operated knee. Secondary outcomes include:

- Pain and physical functioning: OKS [35]
- Neuropathic pain: PainDETECT [40] and DN4 [41]
- Psychological status: HADS [39], Pain Catastrophizing Scale [46], and Possible Solutions to Pain Questionnaire [47]
- Use of pain medications: Resource use questions (to be analysed as part of the cost-effectiveness analysis)
- Improvement and satisfaction with pain relief: Self-Administered Patient Satisfaction Scale [48], single-item question on comparison of pain to pre-operative pain
- Temporal aspects of pain: Single-item questions on pain frequency during past 24 h and 4 weeks.
- Capability: ICECAP-A [49]
- Health-related quality of life: EQ-5D-5 L [50] and Short Form-12 [51]
- Pain elsewhere: body diagram to assess chronic widespread pain [52]

Mean/median scores at 12 months post randomisation will be analysed for continuous outcomes. The HADS and the single-item questions on pain frequency and comparison to pre-operative pain will be analysed as ordinal variables. Pain elsewhere will be treated as a dichotomous outcome, with patients defined as having chronic widespread pain if they report pain in at least two sections of each two contralateral limbs and in the axial skeleton.

The final questionnaire includes free-text questions that ask participants to explain what has and has not helped with their knee pain over the duration of the trial.

### Resource use

Resources used in relation to the intervention (including initial face-to-face assessment and telephone contacts) will be recorded on a standardised proforma. Use of health services including primary, secondary and tertiary care, use of personal social services and additional costs (private healthcare, travel, lost income, home modifications) will be collected in the follow-up questionnaires at 6 and 12 months after randomisation. Participants are provided with resource diaries and prescribed medication folders to prospectively record and document any health resources they have used to assist them in the completion of the questionnaires [53]. Resource use data, including inpatient stays and outpatient visits for all participants at the treating hospitals, will be obtained from hospital electronic systems or extracted from hospital records and recorded on a standardised proforma.

### Internal pilot phase

The 6-month internal pilot phase at four trial sites will evaluate patient identification and eligibility, recruitment rates, withdrawal rates and reasons for withdrawal, questionnaire completion rates, adverse reactions and protocol compliance. Embedded qualitative research, involving audio-recording of recruitment consultations and telephone interviews with participants, will be undertaken to optimise recruitment and trial processes. Anonymised transcripts from the recruitment consultations and interviews will be imported into the qualitative data management software QSR NVivo™. Data will be analysed thematically, involving inductive and deductive coding and categorisation [54]. Data from the pilot phase will be used to inform refinements to recruitment and trial processes. Patients recruited into the pilot phase will continue with the follow-up schedule and be retained in the full trial analysis.

### Safety

Data on adverse events reactions (adverse events directly attributable to the intervention) are collected and closely monitored to ensure the ongoing safety of participants. All serious adverse events will be notified to the study sponsor and reviewed by the Trial Steering Committee.

### Withdrawals

Participants are free to withdraw from the trial at any point. All withdrawals will be recorded on a standardised form. Those who withdraw from the trial will be asked if they would be willing to discuss their reasons for withdrawal to allow the identification of any barriers to participation and highlight whether measures to facilitate participation in the trial need to be implemented.

### Qualitative study

After the 12-month follow-up, a purposive sample of up to 30 participants from the intervention group will be interviewed about the STAR care pathway. This sample size should be sufficient to achieve data saturation in keeping with standards of qualitative research [55, 56]. Interviews will address participants’ experiences of the pathway and their experience of surgery, pain, and resource use. With participants’ consent, interviews will be audio-recorded and anonymised transcripts imported into QSR NVivo™ and analysed using a thematic approach [54]. Findings will be used to further inform the interpretation of the trial’s findings as well as implementation into clinical practice.

### Thank you cards and newsletters

Cards will be sent to participants at 3 and 9 months after randomisation to thank them for their continuing involvement in the STAR trial and to remind them when they can expect to receive the next STAR questionnaire. Newsletters will be sent to all participants every 6–12 months to keep them updated on trial progress.

### Sample size

For a 2:1 intervention:control randomisation ratio, a sample size of 285 patients would have a power of 80% to 90% to detect standardised differences of between 0.35 and 0.40 standard deviations using a two-sided 5% significance level. From previous studies [57, 58], the standard deviations for each of the BPI Interference and Pain Severity scales for patients with chronic post-surgical pain have been observed to be approximately 2, in which case, the target effect size translates to a difference between intervention and control groups of between 0.7 and 0.8 scale points for both scales. Such a difference is worthwhile detecting clinically, since the current consensus statement indicates that differences of approximately one scale point can be deemed the minimally important difference for both of these scales [58, 59]. To allow for a conservative 25% loss to follow-up in the STAR trial, 381 participants will be recruited.

### Data management

Participants’ personal data will be regarded as strictly confidential and will be entered onto a secure administrative Microsoft Access™ database stored on a University of Bristol server. Only STAR team members with appropriate contracts/letters of access with NHS trusts will have access to participants’ personal data. Anonymised trial data will be stored using REDCap, an online secure application [60]. REDCap will also be used to administer online questionnaires to trial participants. Double data entry of the primary outcome measure for all participants completing paper questionnaires and full Case Report Forms for a random sample of participants will be undertaken to ensure data quality.

### Data monitoring

The trial will be overseen by an independent Trial Steering Committee (TSC), composed of four clinical or non-clinical academics and one member of the public. The TSC will meet at regular intervals to review trial progress, protocol adherence and patient safety. The TSC decided that a Data Monitoring Committee was not necessary for this trial and that safety data and data quality will be reviewed by the TSC. No formal interim analysis will be conducted; however, data from the pilot phase were analysed to evaluate the feasibility of proceeding to the main trial. The trial will be stopped earlier than planned if mandated by the NHS Ethics Committee, recommended by the TSC, funding for the trial ceases or for any other relevant major clinical or therapeutic reason.

### Auditing

The coordinating centre will regularly monitor trial sites to ensure data quality and completeness. The trial sponsor (North Bristol NHS Trust) will monitor the trial, potentially including reviewing the Site Files and participants’ medical records.

### Statistical analysis

The full statistical analysis plan for the STAR trial can be accessed at the University of Bristol publications repository [61]. Data analysis will be conducted in accordance with CONSORT guidelines, commencing with descriptive analyses to compare groups at baseline. The primary comparative analysis will apply the intention-to-treat principle including all participants as randomized and with primary outcome data available at 12 months after randomisation. The mean BPI pain severity and BPI interference scores at 12 months after randomisation will be compared between the usual care and intervention groups using linear regression models, adjusting for the respective baseline score and the minimisation/stratification variables. Sensitivity analyses will use standard imputation techniques to impute missing primary outcome data. The secondary outcomes will be analysed using regression models in a similar manner to the primary analysis. Subgroup analyses will investigate variation in the treatment effect between orthopaedic centres and by pain severity, using interaction terms added to the regression models. Explanatory analyses, such as Complier Average Causal Effect methodology, will be used to estimate the effect in those patients able to comply with their allocated intervention. Compliance in the intervention group is defined as attendance at the STAR assessment clinic.

### Cost-effectiveness analysis

The primary cost-effectiveness analysis will take an NHS and Personal Social Services perspective. A secondary analysis will take a broader perspective to include patients’ costs. Only resources used in relation to the treatment of chronic pain will be measured from randomisation to 12 months follow-up. All resources will be valued using routine data sources and information from hospital finance departments. All analyses will be on an intention-to-treat basis and there will be no discounting of costs or effects given the 1 year duration of the study. The primary outcome for the economic evaluation will be the Quality Adjusted Life Year (QALY). The difference in costs and QALYs between the arms will be assessed using the Net Benefit framework using appropriate regression models adjusted for baseline values of the minimisation/stratification variables. Additionally, the difference in costs and those in primary outcomes will be examined. If no arm is dominant, then incremental cost-effectiveness ratios will be calculated using, if appropriate, Seemingly Unrelated Regressions to account for the potential correlation between costs and the primary outcomes. Given the number of important secondary outcomes, a cost consequence analysis will also be conducted in relation to these outcomes. Uncertainty will be addressed using cost-effectiveness acceptability curves and sensitivity analyses.

### Dissemination policy

Publications will include a final report, presentations at scientific meetings and open-access articles in peer-reviewed journals. Avenues for disseminating findings to patients and the public will be identified and developed in collaboration with the PEP-R patient involvement groups and relevant charity organisations such as Arthritis Care. In addition, all participants who indicate that they wish to receive study results will be sent a plain English summary of the final results.

## Discussion

To our knowledge, this is the first randomised controlled trial to evaluate the clinical and cost-effectiveness of a care pathway when compared with usual care for patients screened as having early indications of chronic pain after total knee replacement. The care pathway aims to identify the potential causes of pain to enable early appropriate onwards referral to existing services for targeted and individualised treatment to improve pain management and to reduce the impact of pain. The design and development of this complex intervention has been informed by multiple stages of work [30], in line with Medical Research Council guidance on the development of complex interventions [62].

There are practical and operational issues pertinent to this trial, particularly regarding screening and randomisation of patients. Approximately 1 in 5 patients experience chronic pain after total knee replacement and therefore this trial involves a stage of screening to identify this subgroup early in the postoperative period. An issue is that patients with poorer outcomes after joint replacement are less likely to respond to postal questionnaires [63]. A Cochrane review identified a number of strategies to improve response rates to questionnaires [64], and we have implemented a number of these, including pre-notification screening cards and non-monetary incentives in the form of a teabag to indicate that the study team appreciate that completion of trial questionnaires requires time and effort from the participant.

In this trial, we are randomising patients on a 2:1 intervention:control allocation ratio. Justification for the use of unequal randomisation allocation is often poorly reported [65]. There are numerous reasons given for the use of unequal randomisation ratios, including to reduce costs, improve recruitment, increase the amount of information on the new treatment including safety data, and to account for differential loss to follow-up and cross-over [65–67]. In this trial, randomisation will take place on a 2:1 basis to ensure that the intervention service is running at sufficient capacity to enable a pragmatic assessment of its effectiveness and, particularly, cost-effectiveness. Providing potential participants with an explanation for the reasons behind 2:1 randomisation is important to ensure that equipoise is conveyed adequately. To address this concern, we are undertaking patient involvement activities and qualitative research within the internal pilot phase with the aim of improving the verbal and written information we provide to potential participants about randomisation.

The findings of this trial will provide evidence to guide decisions by clinicians, policymakers and patients, and to inform commissioning of services. If shown to be clinically and cost-effective, this intervention could improve the early identification and management of chronic pain after total knee replacement. It is also possible that this model of care delivery could be adapted for the evaluation of the management of chronic post-surgical pain in other surgical contexts.

### Trial status

The first participant was recruited into the trial in October 2016. Recruitment is scheduled to be completed by March 2019, and follow-up and data collection are scheduled to be completed by March 2020.

## Additional file

Additional file 1: SPIRIT checklist. (DOCX 23 kb)

## Appendix

**Table 1:** World Health Organization Trial Registration Data Set

Item number	Item	Description
1	Primary registry and trial identifying number	ISRCTN92545361
2	Date of registration in primary registry	30/08/2016
3	Secondary identifying numbers	REC reference: 16/SW/0154 NIHR Programme Grant for Applied Research reference: RP-PG-0613-20,001
4	Sources of monetary or material support	NIHR Programme Grant for Applied Research
5	Primary sponsor	North Bristol NHS Trust Research and Innovation, Learning and Research Building, Southmead Hospital, Bristol, BS10 5NB Tel: 0117 414 9330 E-mail: research@nbt.nhs.uk
6	Secondary sponsor	Not applicable
7	Contact for public queries	Wendy Bertram Musculoskeletal Research Unit, Translational Health Sciences, Bristol Medical School, University of Bristol Learning & Research Building, Southmead Hospital, Bristol, BS10 5NB Telephone: 0117 414 7848 E-mail: wendy.bertram@bristol.ac.uk
8	Contact for scientific queries	As above
9	Public title	Evaluation of a care pathway for patients with long-term pain after knee replacement
10	Scientific title	Evaluation of the clinical and cost-effectiveness of a care pathway for patients with chronic pain after total knee replacement: STAR trial
11	Countries of recruitment	UK
12	Health condition(s) or problem(s) studied	Chronic pain after total knee replacement
13	Intervention(s)	Intervention group: One-hour STAR assessment clinic with an Extended Scope Practitioner to identify potential causes of pain and enable onwards referral to appropriate existing services. Up to 6 telephone follow-up calls from the Extended Scope Practitioner over the 12 month follow-up period. Control group: Care as usual
14	Key inclusion and exclusion criteria	Age: 18 years or over; no upper age restriction Sex: Male or female Inclusion: Patients who have received a primary total knee replacement because of osteoarthritis at a participating NHS Trust and who report pain in their operated knee at 2–3 months after surgery (score of 0–14 on the Oxford Knee Score pain subscale). Exclusion: A lack of capacity to provide informed consent to participate, previous participation in the STAR trial for the contralateral knee, participation in another research study that interferes unacceptably with the STAR trial
15	Study type	Interventional Allocation: Randomised Assignment: parallel Phase III
16	Date of first enrolment	October 2016
17	Target sample size	381
18	Recruitment status	Recruiting
19	Primary outcomes	Pain intensity and pain severity at 12 months after randomisation, measured using the Brief Pain Inventory
20	Key secondary outcomes	Brief Pain Inventory at 3 and 6 months after randomisation Oxford Knee Score at 3, 6 and 12 months after randomisation painDETECT at 3, 6 and 12 months after randomisation Douleur Neuropathique 4 at 3, 6 and 12 months after randomisation Hospital Anxiety and Depression Scale at 3, 6 and 12 months after randomisation Pain Catastrophizing Scale at 3, 6 and 12 months after randomisation Possible Solutions to Pain Questionnaire at 3, 6 and 12 months after randomisation Self-Administered Patient Satisfaction Scale at 3, 6 and 12 months after randomisation ICECAP-A at 3, 6 and 12 months after randomisation EQ-5D-5 L at 3, 6 and 12 months after randomisation SF-12 at 3, 6 and 12 months after randomisation

## Abbreviations

BPI: Brief Pain Inventory
DN4: Douleur Neuropathique 4
GP: General Practitioner
HADS: Hospital Anxiety and Depression Scale
ISRCTN: International Standard Randomised Controlled Trial Number
NHS: National Health Service
OKS: Oxford Knee Score
PEP-R: Patient Experience Partnership in Research
QALY: Quality Adjusted Life Year
SPIRT: Standard Protocol Items: Recommendations for Interventional Trials
STAR: Support for Treatment After joint Replacement
TSC: Trial Steering Committee
